# Health conspiracy theories: a scoping review of drivers, impacts, and countermeasures

**DOI:** 10.1186/s12939-025-02451-0

**Published:** 2025-04-03

**Authors:** Adnan Kisa, Sezer Kisa

**Affiliations:** 1 School of Health Sciences, Kristiania University of Applied Sciences, Oslo, Norway; 2 Department of International Health and Sustainable Development, Celia Scott Weatherhead School of Public Health and Tropical Medicine, Tulane University, New Orleans, U.S.A.; 3 Faculty of Health Sciences, Oslo Metropolitan University, Oslo, Norway

**Keywords:** Health equity, Health conspiracy theories, Health literacy, Marginalized groups, Trust in healthcare, Misinformation mitigation

## Abstract

**Background:**

Health-related conspiracy theories undermine trust in healthcare, exacerbate health inequities, and contribute to harmful health behaviors such as vaccine hesitancy and reliance on unproven treatments. These theories disproportionately impact marginalized populations, further widening health disparities. Their rapid spread, amplified by social media algorithms and digital misinformation networks, exacerbates public health challenges, highlighting the urgency of understanding their prevalence, key drivers, and mitigation strategies.

**Methods:**

This scoping review synthesizes research on health-related conspiracy theories, focusing on their prevalence, impacts on health behaviors and outcomes, contributing factors, and counter-measures. Using Arksey and O’Malley’s framework and the Joanna Briggs Institute guidelines, a systematic search of six databases (PubMed, Embase, Web of Science, CINAHL, PsycINFO, and Scopus) was conducted. Studies were screened using predefined inclusion and exclusion criteria, with thematic synthesis categorizing findings across diverse health contexts.

**Results:**

The review revealed pervasive conspiracy beliefs surrounding HIV/AIDS, vaccines, pharmaceutical companies, and COVID-19, linked to reduced vaccine uptake, increased mistrust in health authorities, and negative mental health outcomes such as anxiety and depression. Key drivers included sociopolitical distrust, cognitive biases, low scientific literacy, and the unchecked proliferation of misinformation on digital platforms. Promising countermeasures included inoculation messaging, media literacy interventions, and two-sided refutational techniques. However, their long-term effectiveness remains uncertain, as few studies assess their sustained impact across diverse sociopolitical contexts.

**Conclusion:**

Health-related conspiracy theories present a growing public health challenge that undermines global health equity. While several interventions show potential, further research is needed to evaluate their effectiveness across diverse populations and contexts. Targeted efforts to rebuild trust in healthcare systems and strengthen critical health literacy are essential to mitigate the harmful effects of these conspiracy beliefs.

## Introduction

Conspiracy theories have become a significant barrier to global health efforts. They erode public trust, promote questionable health behaviors, and widen health disparities. These theories often stem from historical injustices, political ideologies, and social anxieties, and their impact has been amplified by digital communication platforms. For example, beliefs about HIV/AIDS being a sinister tool of oppression [1, 2] or claims that vaccines are deliberately designed to cause infertility or autism [3, 4] have contributed to reduced health-seeking behaviors and increased vaccine hesitancy.

The consequences of these health-related conspiracy theories are wide-ranging. They influence psychological, social, and behavioral outcomes. They discourage participation in biomedical research [2], reduce vaccine uptake [4, 5], and are associated with increased anxiety and depression [6, 7]. Marginalized communities are particularly vulnerable, as conspiracy beliefs exacerbate existing health inequities and limit access to accurate health information and care. These disparities are often compounded by systemic factors such as educational inequalities, socioeconomic barriers, and cultural mistrust of health systems [1, 8–10]. Understanding these effects is crucial for designing targeted interventions that can counteract the negative influence of conspiracy theories.

The spread of conspiracy theories is driven by multiple factors, including individual psychological tendencies, social structures, and systemic distrust. Cognitive biases, such as susceptibility to fear-based messaging and low levels of analytical thinking, increase the likelihood of adopting conspiratorial beliefs [11, 12]. Additionally, structural factors, such as political instability, media misinformation, and reliance on non-credible sources, have facilitated the dissemination of these narratives [13, 14]. Digital platforms have further accelerated the spread, with social media algorithms prioritizing engaging but unverified content, as seen during the Zika outbreak and COVID-19 pandemic [14, 15]. Social media platforms such as Facebook, YouTube, and Twitter have been found to amplify misinformation by prioritizing sensational content that garners high engagement [15]. During the COVID-19 pandemic, false claims about vaccines and treatments gained traction through viral videos, automated bots, and coordinated disinformation campaigns [14, 15]. Similarly, search engines and video recommendation algorithms contribute to misinformation exposure by steering users toward unverified or conspiratorial content over time [15]. These factors make conspiracy theories highly accessible and reinforce users’ pre-existing beliefs [15–18].

Efforts to address these conspiracy theories have yielded varied effectiveness, influenced by the type of intervention and contextual factors. Educational interventions, such as inoculation messages and media literacy programs, have shown promise in reducing susceptibility to misinformation, but their long-term impact remains uncertain, particularly in digital spaces where new conspiratorial content continuously emerges [16, 17]. Similarly, strategies aimed at enhancing trust in health institutions and promoting analytical thinking have demonstrated effectiveness, but their success often depends on sociopolitical context, cultural beliefs, and pre-existing mistrust in authorities [18, 19]. Research suggests that even well-designed interventions may require ongoing reinforcement to sustain their impact, as exposure to misinformation can erode prior corrective efforts over time [8, 20]. These challenges highlight the need for adaptive and localized approaches that consider the broader ecosystem in which conspiracy theories spread.

Given the growing influence of health-related conspiracy theories, this scoping review aims to systematically map and synthesize existing research by addressing four critical questions:

1. What are the most common conspiracy theories in the health sector?
2. How do conspiracy theories influence health behaviors, attitudes, and outcomes?
3. What factors contribute to the spread of conspiracy theories in the health sector?
4. What strategies have been proposed or implemented to address conspiracy theories in healthcare?

This is the first scoping review to provide a structured synthesis of research on health-related conspiracy theories across multiple disciplines. By integrating findings from diverse methodologies, as well as including cross-sectional studies, experiments, and content analyses, this review offers a comprehensive framework for understanding the impact of conspiracy theories on public health. Additionally, it highlights the critical link between conspiracy beliefs and health equity, emphasizing the need for strategies that address disparities in health outcomes and access to care. The insights gained from this analysis will support the development of evidence-based interventions and policy recommendations aimed at reducing the harm of conspiracy beliefs and strengthening trust in health systems.

## Methodology

### Design

This scoping review systematically explored the literature on health-related conspiracy theories. It followed the framework outlined by Arksey and O’Malley (2005) [19], as further refined by Levac et al. (2010) [20] and the Joanna Briggs Institute (JBI) guidelines. The scoping review approach is designed to map existing evidence comprehensively, identify research gaps, and provide an overview of the key findings in this emerging area. To ensure methodological rigor and relevance, this study relied exclusively on primary research articles.

### Search strategy

The search strategy was developed to systematically identify primary research articles from six major databases: PubMed, Embase, Web of Science, CINAHL, PsycINFO, and Scopus. Boolean operators were used in the search query: (“conspiracy theory” OR “misinformation” OR “health rumors” OR “health myths”) AND (“healthcare” OR “public health” OR “medical sector”). The search included all English-language publications available up to October 30, 2024. The terms and structure of the search were tailored to each database’s specific indexing vocabulary, ensuring precision and inclusiveness. Duplicate records were removed using EndNote software, and only peer-reviewed primary research articles were considered for inclusion.

### Inclusion and exclusion criteria

To maintain a focused scope, the review applied specific inclusion and exclusion criteria. Included studies addressed conspiracy theories related to health, healthcare, or public health campaigns, and explored their prevalence, impacts, contributory factors, or mitigation strategies. Studies employing any primary research design (quantitative, qualitative, or mixed-methods) were included. Excluded studies were those having topics unrelated to health, published in non-English languages, or classified as editorials, opinion pieces, conference abstracts, dissertations, or secondary analyses.

### Study selection and data extraction

**Fig. 1:**
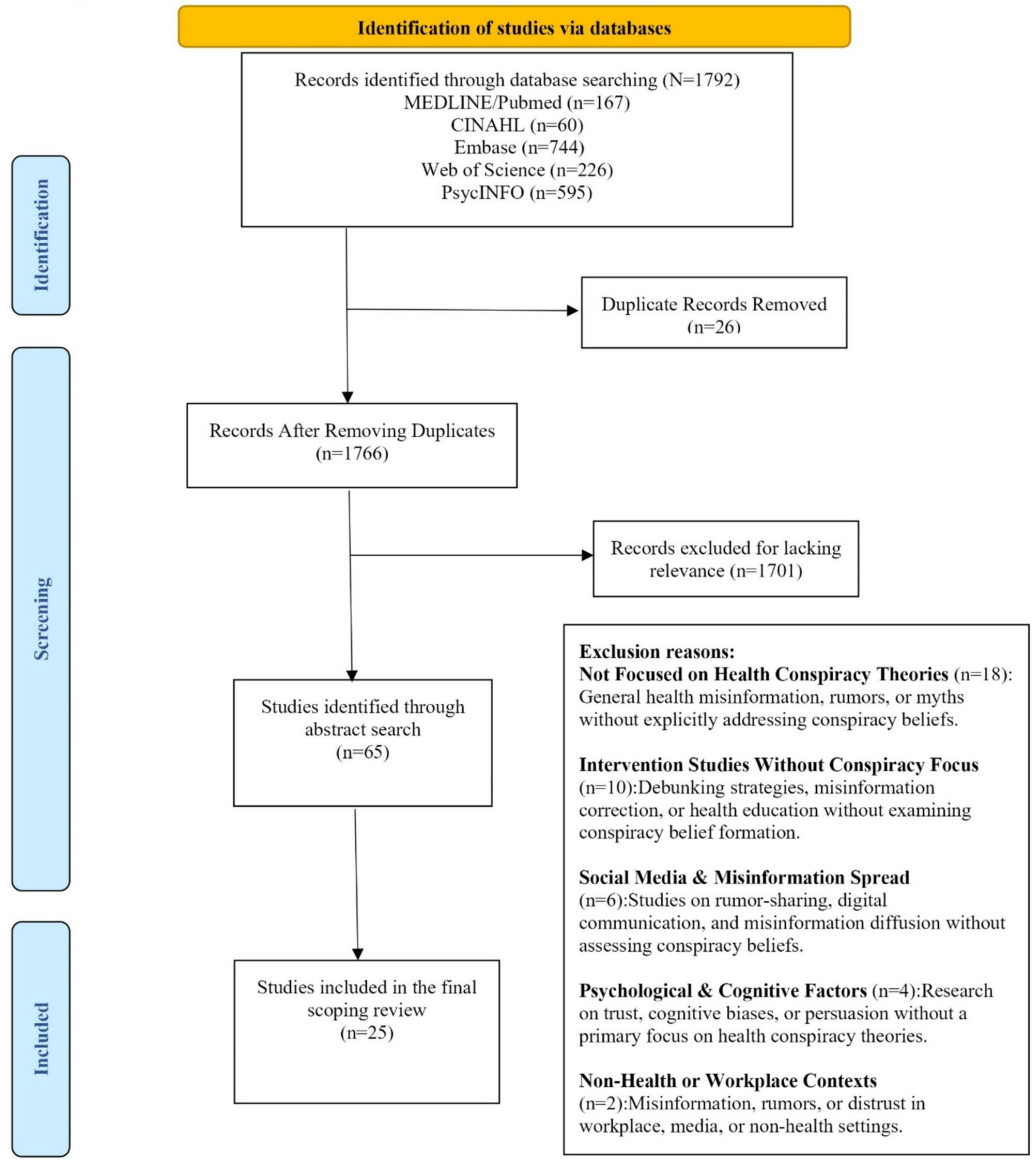
PRISMA

Two reviewers independently screened the titles and abstracts of the identified studies to ensure they met the inclusion criteria. Full-text screening followed for studies deemed potentially relevant. Discrepancies were resolved through discussion between the reviewers. A standardized data extraction form was developed collaboratively to capture essential information, including authorship, publication year, country, study design, and key findings. The extraction process focused on answering the predefined research questions related to the prevalence, influence, contributing factors, and mitigation strategies of health-related conspiracy theories. Figure 1 presents the PRISMA diagram of the study selection process.

### Data synthesis

Thematic analysis was conducted to synthesize data from the included studies. The findings were organized according to the research questions, highlighting patterns and trends in the literature. Results are presented in narrative and tabular formats to summarize the prevalence and impacts of conspiracy beliefs, the factors contributing to their spread, and the strategies for combatting them.

## Results

### Characteristics of the studies

Twenty-five included studies examined various aspects of health-related conspiracy theories by focusing on their prevalence, psychological impact, and influence on health behaviors and public trust (Table 1). Early research explored AIDS-related conspiracy beliefs and their effects on biomedical research participation [2], government mistrust, and HIV testing [1], including knowledge among South African adolescents [21]. Broader conspiracy theories and their psychological associations were also analyzed, particularly links between Medical Conspiracy Theories (MCTs), which involve distrust in healthcare systems and medical interventions, and Modern Health Worries (MHWs), which reflect fears about environmental and technological health risks [24].Studies investigated the spread of Zika-related rumors [14], vaccine misinformation [3], and the role of social media in amplifying misinformation. The COVID-19 pandemic intensified research into conspiracy beliefs among healthcare workers [6], their association with mental health outcomes [7], political ideology [13], and social determinants like trust and ideological orientation [8]. Additionally, interventions such as infographics to improve trust in science [22], fact-based and logic-based inoculation messages [15, 23], and media literacy strategies were assessed for effectiveness in countering conspiracy beliefs [4, 17]. Other studies explored theoretical predictors, including the Theory of Planned Behavior (TPB), which suggests that vaccine hesitancy is influenced by attitudes, perceived social norms, and perceived behavioral control, and the Health Belief Model (HBM), which posits that individuals’ decisions about vaccines depend on perceived susceptibility to disease, perceived benefits, and barriers to vaccination [26]. Additionally, research examined the role of influencers in spreading misinformation through parasocial relationships and gendered narratives, which shape public perceptions of vaccine risks and benefits [27].

Study characteristics varied widely in sample size, setting, and target populations. Sample sizes ranged from 165 students [23] to large-scale surveys with 45,772 participants across 66 countries [16]. Research settings included online surveys [17, 22, 24], hospital-based studies in Ecuador [6], and community-based studies in South Africa [21]. Analysis of social media helped in understanding the dissemination of misinformation [13, 18, 25].

The included studies covered diverse populations, from healthcare workers [6] and adolescents [21] to general adult populations across different countries [5, 10]. Additionally, some studies focused on niche online communities, such as White nationalist forums and wellness influencers, to explore ideological and commercial motivations behind misinformation [18, 25].

**Table 1:** Characteristics of included studies

Author(s), year, country	Study design	Aim	Sample size	Setting	Population	Findings
Russell et al., 2011, USA [2]	Cross-sectional	Examine the belief in AIDS origin conspiracy and its relationship to participation in biomedical research.	1,133 (1999–2000) & 1,162 (2003)	4 US cities (1999–2000), 3 US cities (2003)	Black, Hispanic, and White adults	27.8% of Blacks and 23.6% of Hispanics believed the AIDS origin story in 1999–2000, increasing to 34.1% and 21.9% in 2003. Belief did not reduce likelihood of research participation.
Ford et al., 2013, USA [1]	Cross-sectional	Examine relationships between belief in AIDS-related stories, government mistrust, and HIV testing.	226	Public health venues	At-risk older adults (≥ 50)	30% believed in AIDS-related stories; 72% mistrusted the government. Conspiracy beliefs increased odds of HIV testing, while government mistrust decreased them.
Hogg et al., 2017, South Africa [21]	Cross-sectional observational	To examine adolescents’ knowledge about HIV origins and conspiracy beliefs.	830	Soweto, South Africa	Adolescents aged 14–19 years	8.6% believed in theories about HIV origins. Conspiracy beliefs were more common among men, unemployed, and those with a relative who died of HIV. Accurate knowledge of HIV origins was associated with being male, older, and unemployed.
Lahrach & Furnham, 2017, UK [24]	Cross-sectional survey	Examine the relationship between MCTs and MHWs, alongside other factors.	335	Online (social media, AmazonTurk)	General British public	MCTs strongly predicted higher MHWs, with significant associations observed across demographics, health perceptions, and CAM usage.
Sommariva et al., 2018, USA [14]	Mixed-methods content analysis	Explore the spread of health rumors and verified information on social networking sites (SNSs) using the Zika virus as a case study.	120 news stories	SNSs	General public engaged on SNSs	Popular rumors portrayed the Zika virus as a conspiracy, a low-risk issue, and connected it to pesticide use. Rumors had three times more shares than verified stories.
Featherstone & Zhang, 2020, USA [3]	Online survey experiment	Examined the short-term effects of vaccine misinformation and refutational messages on attitudes and emotions.	609	Online (MTurk)	US adults	Conspiracy-framed misinformation decreased pro-vaccination attitudes by evoking anger; refutational messages mitigated these effects.
Chen et al., 2020, Ecuador [6]	Cross-sectional survey	Investigate belief in COVID-19 theories as predictors of mental health and wellbeing among healthcare workers.	252	Hospitals, clinics, nursing homes, dental clinics, pharmacies	Healthcare workers	24.2% believed COVID-19 was developed in a lab. Conspiracy belief linked to higher anxiety, distress, and lower job and life satisfaction.
Havey, 2020, USA [13]	Sentiment analysis of Twitter data	Investigate how political ideology influences support for COVID-19 misinformation and conspiracy theories.	4101 tweets	Twitter	Twitter users discussing six misinformation topics related to COVID-19	Conservatives dominate misinformation discourse, promoting conspiracies about Bill Gates, the Chinese Communist Party, and the Deep State. Misinformation impacts adherence to public health recommendations.
Agley et al., 2021, USA [22]	Randomized Controlled Trial (RCT)	Examine if an infographic about the scientific process can increase trust in science and reduce belief in misinformation.	1017	Online (Prolific platform)	US representative sample of adults by age, race/ethnicity, and gender	Brief exposure to the infographic increased trust in science slightly and reduced belief in COVID-19 misinformation indirectly through trust in science.
Loomba et al., 2021, UK & USA [4]	Randomized Controlled Trial (RCT)	Quantify how exposure to online misinformation affects intent to vaccinate.	8,001	Online panel survey	Nationally representative samples of UK and US populations by gender, age, and region	Exposure to misinformation decreased intent to vaccinate by 6.2% in the UK and 6.4% in the USA, compared to factual information.
Natoli & Marques, 2021, Australia [11]	Randomized experimental (3 studies)	Investigate how antidepressant conspiracy theories impact health-seeking intentions and explore trust and powerlessness as mediators.	299, 244, 247	Online (MTurk, Facebook)	General population (USA and Australia)	Exposure to conspiracy theories decreased health-seeking intentions indirectly by reducing trust in health authorities. Powerlessness had mixed effects.
Juanchich et al., 2021, UK [12]	Cross-sectional	Examine the link between COVID-19 beliefs and health protective behaviors.	302, 404, 399	UK	General population	COVID-19 conspiracy beliefs correlated with higher mistrust in government, reduced intention to vaccinate, and increased misinformation sharing.
Dȩbski et al., 2022, Poland [7]	Cross-sectional survey	Examine the relationship between belief in COVID-19 theories and symptoms of anxiety and depression.	700	Online survey	Adult Poles (mean age 24.8 ± 6.3, 83.6% female)	Positive correlation between belief in COVID-19 conspiracy theories and symptoms of anxiety (*r* = 0.087) and depression (*r* = 0.108).
McCarthy et al., 2022, Australia [5]	Cross-sectional survey	Examine how conspiracy beliefs, perceived health threats, trust in government, and anomie mediate COVID-19 vaccine hesitancy.	779	National online survey via Facebook	Australian adults	Belief in conspiracy theories predicts vaccine hesitancy through mediators such as perceived health threats and anomie.
Jiang et al., 2022, Hong Kong [15]	Three-phase experiment	Test the effectiveness of inoculation messages against COVID-19 misinformation in a low political trust context.	123	University students	College students aged 17–26	Inoculation messages led to more positive vaccine attitudes and stronger vaccine intentions compared to supportive and control messages. Low political trust facilitated misinformation spread.
Swire-Thompson et al., 2023, USA [26]	Longitudinal experimental study	Examine the association between memory for corrections and belief regression after correction.	612	Prolific Academic	General population	Memory plays a fundamental role in belief regression. Repeated corrections can be an effective strategy to counteract belief regression.
Lin et al., 2023, multinational (66 countries) [16]	Cross-sectional multinational survey	Examine the role of risk perception and conspiracy theory endorsement on compliance with COVID-19 public health measures, moderated by age and country development.	45,772	Online survey during early COVID-19 pandemic	Adults (18–100 years old) from various countries	Higher conspiracy theory endorsement correlated with lower compliance with public health measures. Conspiracy theories influenced the perception of risk and trust in health measures, particularly in developed countries.
Nefes et al., 2023, Spain [8]	Cross-sectional survey	Examine social factors associated with belief in conspiracy theories during COVID-19 in Spain.	2100	Nationwide	Spanish residents aged 18+	Beliefs associated with worse vaccination behaviors. Weak association with demographics (age, sex, education). Related to trust (instrumental rationality) but less so to value rationality. Ideological orientation correlates with conspiracy beliefs. Religious affiliation had minimal influence.
Walter et al., 2023, USA [25]	Mixed-method study	To examine White nationalist discourse on COVID-19 and its containment measures in an online forum.	9270 posts	Online (Stormfront)	White nationalist online community	Identified four themes in discourse (Science, Conspiracies, Sociopolitical, Containment) and revealed high levels of misinformation and conspiracy theories about COVID-19.
Banas et al., 2024, USA [23]	Independent-group experiment	Examine fact- and logic-based inoculation treatments in preventing anti-vaccination propaganda.	165	University setting, USA	College students, aged 18–28	Both fact-based and logic-based inoculations were effective in instilling resistance to anti-vaccination conspiracy propaganda. Resistance did not cross-protect against other conspiracies.
Kapoor et al., 2024, India [10]	Cross-sectional survey	Examine psychosocial predictors of beliefs in health-related misinformation	826	Online	Indian participants aged 18–71	Lower socioeconomic status, lower trust in political institutions, negative moral emotions, right-leaning ideology, and conspiratorial thinking predict belief in health-related conspiracies
Lyons et al., 2024, USA [17]	Experimental survey	Test the effects of health-focused vs. generic media literacy interventions on cancer news evaluation.	1,200	Online survey (US national sample)	US adults (median age 53)	Health-focused intervention increased skepticism of both accurate and inaccurate news. Generic intervention improved sharing discernment, reducing sharing of inaccurate news but without significant effects on perceived accuracy. False cancer beliefs were key in susceptibility to conspiracy beliefs.
Carletto et al., 2024, USA [9]	Cross-sectional survey	Examine the associations of sociodemographic characteristics, health literacy, and mistrust with COVID-19 conspiracy and health myths.	561	Primary care clinics in Maryland and Pennsylvania	Adults with hypertension (mean age 62.3 years, 60.2% female, 46% Black, 10.2% Hispanic)	Lower education, health literacy, higher mistrust, and reliance on non-professional information sources were associated with belief in conspiracy myths.
Kroke & Ruthig, 2024, USA & Canada [27]	Cross-sectional survey	Test integrated models of the TPB and HBM to determine how beliefs predict vaccination behaviors.	529	USA & Canada	Adults, including undergraduate students and community members	COVID-19 conspiracy beliefs negatively influenced vaccination attitudes, perceived behavioral control, and subjective norms, as well as HBM factors such as susceptibility, severity, benefits, and barriers.
Moran et al., 2024, USA [18]	Digital ethnography	Explore how wellness influencers spread vaccine misinformation on Instagram and leverage it for profit.	3 influencers	Instagram and linked sites	Influencers in the wellness sector	Parasocial relationships and gendered narratives facilitate the spread of vaccine-related misinformation. Monetization of misinformation occurs through affiliate links and product promotions.

### Key findings related to conspiracy theories

Conspiracy theories influence health behaviors, attitudes, and public trust across various contexts. Early studies reported significant belief in AIDS-related conspiracies, particularly among marginalized groups, with 27.8% of Black and 23.6% of Hispanic participants endorsing the AIDS origin conspiracy in 1999–2000, increasing to 34.1% and 21.9% in 2003 [2]. Among older adults (≥ 50 years), 30% believed in AIDS-related conspiracy theories, which increased HIV testing likelihood but also reduced trust in government [1]. In South Africa, 8.6% of adolescents believed in HIV-related theories, with prevalence higher among men, the unemployed, and those who had lost relatives to HIV [21]. COVID-19-related rumors had widespread psychological, behavioral, and societal effects, with 24.2% of healthcare workers in Ecuador believing COVID-19 was created in a lab, a belief associated with higher anxiety, distress, and lower job and life satisfaction (*p* < 0.05) [6]. Similar associations between conspiracy beliefs and anxiety (*r* = 0.087, *p* < 0.05) or depression symptoms (*r* = 0.108, *p* < 0.05) were found in Poland [7]. In Spain, conspiracy beliefs correlated with lower vaccination rates, (*r* = -0.42, *p* < 0.001) particularly among individuals with extreme-right ideological orientations [8]. Studies across 66 countries showed that stronger endorsement of conspiracy theories was linked to lower compliance with public health measures (β = -0.37, *p* < 0.01), with a greater impact in developed nations [16]. In the UK, government mistrust fueled by conspiracy beliefs reduced vaccine intentions [12], while in Australia, perceived health threats and anomie mediated vaccine hesitancy [5]. Exposure to online misinformation reduced vaccination intent by over 6% in the UK and the USA [4].

Efforts to counter conspiracy theories showed mixed results. Brief exposure to an infographic explaining the scientific process increased trust in science and reduced misinformation belief indirectly [25]. Inoculation messages promoting vaccine acceptance were most effective in low-trust settings [17]. Fact-based and logic-based inoculation treatments reduced susceptibility to anti-vaccination propaganda, though they did not protect against other conspiracy beliefs [16]. Media literacy interventions increased skepticism toward both fake and real news, showing potential unintended consequences [18]. Social media platforms played a central role in amplifying conspiracy theories. During the Zika virus outbreak, rumors were shared three times more often than verified information [14]. On Twitter, misinformation discourse was dominated by conservative users, who frequently spread conspiracy theories about Bill Gates, the Chinese Communist Party, and the Deep State [13]. On Instagram, wellness influencers leveraged parasocial relationships and gendered narratives to spread vaccine misinformation for profit through affiliate links and product promotions [27]. A qualitative analysis of White nationalist forums identified four dominant misinformation themes—scientific skepticism, conspiracy claims, sociopolitical concerns, and containment opposition—contributing to vaccine rejection [15]. Medical conspiracy theories strongly predicted health worries, particularly among individuals with lower education, lower health literacy, and greater reliance on non-professional health information sources [9, 24]. Socioeconomic status, trust in political institutions, and moral emotions significantly influenced belief in health-related misinformation [10]. A study in India found that lower socioeconomic status, right-leaning ideology, and negative moral emotions were key predictors of conspiracy theory endorsement [10]. In the USA and Canada, conspiracy beliefs negatively influenced vaccination attitudes, perceived behavioral control, and subjective norms, particularly through factors outlined in the TPB and the HBM, such as perceived susceptibility, severity, and perceived barriers [26].

### Health conspiracy beliefs

Conspiracy theories in the health sector encompass a broad range of beliefs. They are often rooted in mistrust of governments, pharmaceutical industries, and health systems. The AIDS origin theory claims that HIV was created by the government or pharmaceutical companies as a tool for genocide or population control and that a cure is being withheld [1, 2, 21]. While these beliefs did not reduce participation in biomedical research, they correlated with higher mistrust, particularly among Black and Hispanic populations in the U.S [2]. Similarly, vaccination-related conspiracies, such as claims that vaccines cause autism or are used to hide tracking devices, have persisted for years [23, 24], leading to reduced vaccination uptake and increased preference for complementary and alternative medicine (CAM) [24] (Table 2).

The spread of misinformation during public health crises, such as the Zika virus and COVID-19 pandemic, has further amplified conspiracy beliefs. Zika-related stories included claims that the virus was linked to larvicides and pesticides, while it was also dismissed as a low-risk issue [14]. COVID-19 conspiracy theories ranged from claims that the virus was intentionally developed in a lab, that it was a military weapon, was being spread by 5G networks, or was a hoax for financial or political gain [6, 13, 16, 22]. These beliefs also include extreme narratives, such as the idea that COVID-19 vaccines contain microchips for social control [15, 18].

Mistrust in pharmaceutical companies and governments is a recurring theme in health-related conspiracies. Claims that COVID-19 vaccines alter DNA, cause infertility, or serve as bioweapons reflect deep-rooted fears about vaccine safety and motives [4, 5]. Similarly, the endorsement of alternative medicine and allegations of suppressed natural cures for profit have fueled conspiratorial thinking about antidepressants and cancer treatments [11, 17]. Such narratives contribute to the erosion of trust in established healthcare systems and practices.

Conspiracy theories often intersect with broader sociopolitical themes that reflect public anxieties and ideological divisions. Beliefs about COVID-19 being a bioweapon, an example of government overreach through lockdowns, or that vaccines are tools for authoritarian control exemplify how health crises become platforms for political polarization [7, 8, 25]. Specific claims, such as those associating vaccines with ferrous metals or microchips, highlight how fear and misinformation spread through targeted campaigns and social media [9, 10].

The persistence of these beliefs, even after corrections, emphasizes the challenges of combating misinformation. While repeated corrections can help counteract belief regression, entrenched narratives—such as the idea of COVID-19 being a hoax or a government conspiracy—continue to thrive across diverse populations and regions [26, 27]. Such beliefs not only undermine public health efforts, but they also exacerbate divisions within societies.

**Table 2:** Popular conspiracy theories and their impact

Author(s), year, country	Conspiracy theory	Health domain(s)	Impact on behaviors and outcomes	Health impact(s)
Russell et al., 2011, USA [2]	AIDS as a government tool for genocide	HIV/AIDS	Did not reduce participation in biomedical research but correlated with mistrust among minorities	P, S
Ford et al., 2013, USA [1]	HIV created to eliminate certain groups; government withholding AIDS cure	HIV/AIDS	Increased likelihood of HIV testing among believers	B
Hogg et al., 2017, South Africa [21]	HIV originated from the US government, pharmaceutical industry, vaccines, space, or scientists	HIV/AIDS	Skepticism about HIV prevention and treatment tools	B, S
Lahrach & Furnham, 2017, UK [24]	Vaccination causes autism; fluoridation hides contaminants	Vaccinations, water safety	Increased skepticism towards modern medicine, preference for CAM	B, P
Sommariva et al., 2018, USA [14]	Zika virus linked to larvicides and pesticides causing birth defects; Zika virus portrayed as a low-risk issue	Zika virus	Undermined trust in health authorities; reduced prevention (e.g., mosquito control); reduced risk perception, led to low compliance with protective measures	B, P
Featherstone & Zhang, 2020, USA [3]	Governments and pharmaceutical companies hide vaccine dangers for profit	Vaccination (MMR)	Lowered pro-vaccination attitudes, increased anger and fear	P, B
Chen et al., 2020, Ecuador [6]	COVID-19 was developed in a lab	Mental health and well-being	Higher levels of anxiety and psychological distress, lower life/job satisfaction	P, B
Havey, 2020, USA [13]	Bill Gates using COVID-19 for surveillance; Chinese Communist Party created COVID-19; hydroxychloroquine as a treatment; bleach as a preventative measure; Deep State using COVID-19 to quell opposition	COVID-19	Decreased trust in public health recommendations; xenophobia and misinformation amplified; use of unproven treatments; potential adverse effects; harmful behaviors like ingestion of bleach; political polarization	P, B, S
Agley et al., 2021, USA [22]	COVID-19 was developed as a military weapon; face masks cause oxygen deficiency; 5G networks caused COVID-19 spread	COVID-19	Believability of misinformation reduced by increased trust in science	P
Loomba et al., 2021, UK & USA [4]	COVID-19 vaccines will alter DNA, cause infertility, or are bioweapons	COVID-19 vaccination	Reduced intent to vaccinate by inducing skepticism and fear	B, P
Natoli & Marques, 2021, Australia [11]	Antidepressants are overprescribed, ineffective, and harmful; natural cures are suppressed for profit	Mental health	Decreased intention to seek medical and psychological help	B, P
Juanchich et al., 2021, UK [12]	COVID-19 as a bioweapon; vaccine secrecy; 5G spreading COVID-19	COVID-19 pandemic	Reduced vaccination and testing intent; increased adherence to self-controlled measures like handwashing	B, P
Dȩbski et al., 2022, Poland [7]	COVID-19 is a planned act or political manipulation; restrictions as an attack on freedom	COVID-19	Non-compliance with health regulations, increased skepticism towards vaccination	P. S
McCarthy et al., 2022, Australia [5]	Governments use COVID-19 to limit freedoms; COVID-19 is a biological weapon released by China; COVID-19 vaccines harm or control society	COVID-19 vaccine hesitancy	Reduced vaccine uptake due to distrust in government and increased anomie	B, P, S
Jiang et al., 2022, Hong Kong [15]	Claims of microchips in vaccines, vaccines as profit-making schemes	COVID-19 vaccination	Reduced vaccine acceptance, negative vaccine attitudes, lower intention to vaccinate	B, P, S
Swire-Thompson et al., 2023, USA [26]	Belief regression after correction	Misinformation correction	Belief in misinformation regresses over time, even with correction	Cognitive, B
Lin et al., 2023, multinational (66 countries) [16]	COVID-19 as a bioweapon; hoax for financial gains; authoritarian conspiracy	COVID-19 pandemic	Lower compliance with public health measures (e.g., distancing, mask-wearing); higher skepticism of government and health authorities	B, P
Nefes et al., 2023, Spain [8]	COVID-19 was developed in a lab; vaccines unsafe	COVID-19	Lower vaccination rates among adults and their children; increased vaccine hesitancy	B, S, P
Walter et al., 2023, USA [25]	COVID-19 is a hoax; Jewish vaccine conspiracy; lockdowns as a pathway to totalitarianism	COVID-19, vaccines, containment	Undermined trust in vaccines and public health measures, promoted resistance to containment measures	P, B, S
Banas et al., 2024, USA [23]	Vaccines cause autism	MMR vaccine	Anti-vaccination attitudes, vaccine hesitancy	B, P
Kapoor et al., 2024, India [10]	COVID-19 is a bioweapon, vaccine dangers, alternative medicine endorsement	General health, COVID-19, vaccination	Reduced vaccine uptake, noncompliance with health guidelines, preference for alternative medicine	B, P
Lyons et al., 2024, USA [17]	Cancer-related misinformation (e.g., alternative cures, mistrust of pharmaceuticals)	Cancer misinformation	Increased skepticism of accurate information; reduced ability to discern between accurate and inaccurate headlines	B, P, S
Carletto et al., 2024, USA [9]	COVID-19 was created for biological warfare; vaccine safety is compromised	COVID-19 pandemic	Increased vaccine hesitancy; lower adherence to public health measures	B, P, S
Kroke & Ruthig, 2024, USA & Canada [27]	COVID-19 is a hoax or government conspiracy (e.g., bioweapon, exaggeration of severity)	COVID-19	Lower vaccination uptake and booster willingness; reduced trust in health authorities	B, P, S
Moran et al., 2024, USA [18]	Vaccines contain microchips or heavy metals; vaccines cause harm	Vaccination hesitancy	Increased vaccine hesitancy, reliance on alternative treatments, homeschooling to avoid vaccine mandates	B, S

B: Behavioral. P: Psychological. S: Social

### Conspiracy theories in different health domains

Conspiracy theories have shaped public behaviors across many health domains. In the context of HIV/AIDS, beliefs have included claims that the virus was created by governments or pharmaceutical companies for population control, along with accusations that a cure is being withheld to target specific groups [1, 2]. Similar conspiracy narratives persisted among South African adolescents, who attributed HIV’s origins to the US government, drug companies, or scientists, highlighting deep-rooted mistrust in health authorities [21].

Vaccination and water safety have also been targets of conspiracy theories, with persistent claims linking vaccines to autism and fluoride in drinking water to hidden contaminants. These narratives contribute to vaccine hesitancy and broader distrust in public health initiatives [24]. During the Zika outbreak, misinformation circulated about the virus being linked to larvicides and pesticides as causes of birth defects while simultaneously downplaying its risks, creating widespread public confusion [14]. Similar patterns of distrust emerged around the measles, mumps, and rubella (MMR) vaccine, particularly in the United States, where misinformation has fueled skepticism regarding vaccine safety [3, 23].

The COVID-19 pandemic further amplified conspiracy beliefs on a global scale. Claims varied from COVID-19 being a bioweapon or a fabricated crisis for financial and political gain to allegations that vaccines alter DNA, contain microchips, or serve as tools for mass control [4, 13, 15, 18, 22]. Such narratives have substantially undermined public trust and increased vaccine hesitancy, with studies linking conspiracy beliefs to lower vaccination rates [5, 8, 16]. Additionally, misinformation about alternative COVID-19 treatments, including hydroxychloroquine and bleach as treatment measures, exacerbated health risks and fueled skepticism toward medical guidance [13].

Conspiracy theories have also harmed mental health and well-being. Widespread claims that antidepressants are overprescribed, ineffective, or harmful reflect broader distrust in pharmaceutical companies and contribute to reliance on alternative therapies [11]. Among healthcare workers, conspiracy beliefs about COVID-19 being created in a lab have been associated with higher levels of anxiety and distress, along with lower job and life satisfaction [6].

Misinformation has extended into cancer treatment, with narratives promoting alternative cures and fostering mistrust in conventional medicine, ultimately compromising public understanding of evidence-based interventions and delaying critical treatments [17]. Efforts to combat misinformation have included interventions such as inoculation messages and media literacy programs. However, their effectiveness in addressing deeply ingrained conspiracy beliefs remains inconsistent [23, 26, 27].

### Impact of conspiracy beliefs on health behaviors and outcomes

Conspiracy beliefs often undermine public health efforts and promote distrust in healthcare systems. While AIDS-related conspiracy beliefs did not reduce participation in biomedical research, they highlighted systemic mistrust, particularly among minority populations [2]. Similarly, these beliefs were linked to increased HIV testing but also reinforced skepticism about HIV prevention and treatment, demonstrating their complex and contradictory effects on health behaviors [1, 21]. More broadly, conspiracy theories have fueled skepticism toward modern medicine and increased reliance on complementary and alternative medicine (CAM) [24].

During public health crises, conspiracy theories played a role in eroding trust in health authorities and reducing compliance with protective measures. For instance, misinformation surrounding the Zika virus led to diminished risk perception and lower adherence to mosquito control efforts [14]. Similarly, COVID-19 theories contributed to distrust in public health recommendations, encouraged harmful behaviors such as bleach ingestion, and intensified xenophobia and political polarization [13]. These beliefs also resulted in lower compliance with public health regulations, including mask-wearing and social distancing, and heightened resistance to vaccination programs worldwide [16, 25].

Vaccine hesitancy has emerged as a persistent consequence of conspiracy beliefs, particularly those depicting vaccines as unsafe or as instruments of societal control. Claims that COVID-19 vaccines alter DNA or contain microchips have reduced vaccination intent and contributed to negative attitudes toward vaccines [4, 15]. Additionally, distrust in government and perceived health threats have acted as mediators, further strengthening the link between conspiracy beliefs and vaccine hesitancy [5]. In Spain, for example, conspiracy-driven skepticism influenced vaccination decisions among both adults and their children, leading to lower immunization rates [8].

Beyond vaccination, misinformation has exacerbated psychological distress and deterred individuals from seeking medical or psychological help. Conspiracy believers have reported higher anxiety levels, lower job and life satisfaction, and a greater reluctance to engage with healthcare services [6, 11]. In some cases, misinformation has led individuals to favor self-controlled protective behaviors, such as increased handwashing, over scientifically validated interventions like vaccination and diagnostic testing [12]. Even after being corrected, conspiracy beliefs often persist, reflecting the deep-seated nature of these narratives and their resistance to factual counterarguments [26].

The broader consequences of conspiracy beliefs extend to misinformation about cancer treatments and vaccine mandates. Cancer-related conspiracy theories have fueled skepticism toward pharmaceutical interventions, reducing trust in evidence-based medicine and delaying critical treatments [17]. Likewise, vaccine hesitancy has encouraged the use of alternative therapies and motivated some parents to opt for homeschooling to bypass vaccine requirements, particularly in the US [10, 18].

### Impact of conspiracy beliefs on health

Conspiracy theories have far-reaching effects across behavioral, psychological, social, and cognitive domains. They can shape health outcomes and public attitudes in profound ways.

Psychologically, conspiracy beliefs intensify emotions such as fear, anxiety, and mistrust, often leading to negative mental health outcomes. For instance, healthcare workers in Ecuador who believed that COVID-19 was developed in a lab reported heightened distress and lower job satisfaction [6]. Vaccine-related conspiracy beliefs also fueled skepticism and fear, further eroding trust in health authorities [3, 7]. In Spain, such beliefs were linked to increased anxiety and depression [8]. Broader concerns, including fears of government surveillance and societal control, contributed to significant emotional burdens worldwide [10, 27].

Behaviorally, conspiracy beliefs often lead to non-compliance with health recommendations, vaccine hesitancy, and reliance on unproven treatments. While AIDS-related beliefs did not directly reduce participation in biomedical research, they contributed to skepticism and distrust, particularly among minority populations [2]. Misinformation surrounding COVID-19 vaccines reduced vaccine uptake and adherence to public health measures [4, 5]. In the US, misinformation led to dangerous behaviors, such as drinking bleach as a supposed cure [13]. Similarly, distrust in vaccines contributed to reliance on alternative treatments and refusal of routine immunizations, further compromising public health efforts [11, 18].

Socially, conspiracy beliefs undermine trust in health systems and deepen societal divisions. Public health measures, such as masking, social distancing, and vaccination campaigns, faced resistance due to widespread mistrust fueled by conspiracy narratives [25]. Multinational studies demonstrated that belief in COVID-19 conspiracies significantly lowered compliance with public health guidelines, illustrating a global pattern of eroded collective trust [16]. In Spain, conspiracy beliefs directly contributed to lower vaccination rates and reduced herd immunity, further illustrating their societal ramifications [8]. Additionally, distrust in healthcare institutions and governments acted as a barrier to effective health interventions [9, 17, 26].

Cognitively, conspiracy beliefs impair critical thinking and the ability to distinguish accurate information from misinformation. Media literacy interventions sometimes increased skepticism toward both falsehoods and verified information, complicating efforts to correct misinformation [15, 23]. The phenomenon of belief regression—where false beliefs persist even after correction—demonstrates the enduring cognitive impact of conspiracy narratives on decision-making [26]. Across health domains, these findings highlight the widespread influence of conspiracy beliefs on individual behaviors, mental health, and societal trust [1, 14, 21, 24].

### Factors contributing to the spread of conspiracy theories

The spread of health-related conspiracy theories is influenced by a combination of sociopolitical, psychological, and informational factors. Mistrust in government is a recurring theme, particularly among minority groups with histories of discrimination and systemic inequality [1, 2]. In South Africa, structural factors such as racial oppression, poverty, and HIV-related stigma reinforce skepticism toward health interventions [21]. Other contributors include religious beliefs, the use of CAM, and self-reported poor mental health [24](Table 3).

The role of alternative media and the absence of evidence-based information in early public health messaging contribute to misinformation, as seen during the Zika outbreak [14]. Social media platforms further amplify conspiracy narratives by creating echo chambers that foster distrust in authorities [3, 6]. Political ideology and polarization intensify these beliefs, particularly in discussions surrounding vaccines and public health mandates [13]. Additionally, low trust in science, political conservatism, and the perceived severity of health crises contribute to the persistence of conspiracy theories [22].

A decline in trust toward health authorities, combined with feelings of powerlessness, has been linked to changes in health behaviors [11]. Misinformation spreads more rapidly in environments where individuals rely on social media for news and have lower levels of analytical thinking [12]. Psychological factors, including beliefs in global conspiracies, concerns about information control, and personal well-being anxieties, also play a role in shaping conspiracy beliefs [7]. Factors such as anomie, political ideology, and perceived health threats mediate the relationship between conspiracy beliefs and vaccine hesitancy [5].

Misinformation spreads particularly rapidly in regions with low political trust [15]. Cognitive biases, including memory failures, resistance to corrections, and shallow information processing make misinformation difficult to counteract [26]. The prevalence of social media, trust deficits in institutions, and higher Human Development Index (HDI) scores in developed nations have all been associated with the increasing acceptance of conspiracy theories [16]. In Spain, conspiracy narratives are reinforced by low trust in government health authorities, skepticism toward pharmaceutical companies, and alignment with extreme right-wing political ideologies [8].

The politicization of science and the strategic use of scientific language by extremist groups, including those with White nationalist ideologies, further erode public trust in evidence-based information [25]. Misinformation, emotional narratives, and distrust in public health institutions contribute to vaccine hesitancy and resistance to public health measures [23]. Socioeconomic factors, including low income, right-leaning ideology, negative moral emotions, and mistrust in political institutions, are strong predictors of belief in health-related conspiracies [10]. Additionally, false beliefs about cancer treatments, distrust in experts, and low digital and health literacy increase vulnerability to misinformation [17].

Educational background, medical mistrust, and reliance on non-credible sources further contribute to the spread of conspiracy myths [9]. Sociopolitical distrust, the unchecked spread of misinformation through social media, and a perceived lack of transparency from authorities reinforce the persistence of these beliefs [27]. Furthermore, vaccine-related misinformation is often propagated by wellness influencers through parasocial relationships, platform affordances, institutional skepticism, and gendered narratives [18].

**Table 3:** Factors contributing to the spread of conspiracy theories

Author(s), year, country	Contributing factors	How the factors spread conspiracy theories	Evidence on factors driving conspiracy beliefs
Russell et al., 2011, USA [2]	Mistrust in government, socioeconomic status, racial/ethnic identity	Historical events (e.g., Tuskegee Study) amplify mistrust. Lower education and income increase susceptibility. Higher prevalence of conspiracy beliefs in minority populations.	High belief rates among Blacks (34.1%) and Hispanics (21.9%) vs. Whites (8%). Strong correlation with education and income levels. Beliefs were over three times more prevalent in minorities compared to Whites.
Ford et al., 2013, USA [1]	Historical discrimination, low trust in government	Reinforces mistrust in public health initiatives.	High levels of mistrust (72%) reported in study. Associations between mistrust and reduced testing behavior.
Hogg et al., 2017, South Africa [21]	Sociocultural context, HIV-related stigma and misinformation	Contributes to distrust in biomedical science and treatment. Amplifies fear and acceptance of non-evidence-based explanations.	AIDS denialism under Thabo Mbeki’s leadership delayed ART roll-out, fostering distrust. Lack of accurate knowledge among adolescents in Soweto.
Lahrach & Furnham, 2017, UK [24]	Religiousness, CAM use, perceived poor mental health	Higher religiousness and CAM use correlated with MCT belief. Poorer mental health increases vulnerability to conspiracy beliefs.	Correlations between these factors and MCT belief ranging from 0.13 to 0.49.
Sommariva et al., 2018, USA [14]	Popularity of alternative media sources, lack of evidence-based information	Alternative media outlets had the highest reach and share rate for rumors. Initial lack of verified content allowed rumors to dominate the conversation.	66% of popular news stories originated from alternative media. Rumors shared more widely than verified stories. WHO launched fact-checking efforts late in the process.
Featherstone & Zhang, 2020, USA [3]	Social media amplification, distrust in authorities	Frames that evoke distrust (e.g., government collusion) resonate emotionally and amplify misinformation. Social media bots and trolls manipulate narratives to promote anti-vaccine messages.	Bots and trolls on social media promoted anti-vaccine messages to foster skepticism. Misinformation content had higher engagement rates than verified health information.
Chen et al., 2020, Ecuador [6]	Social media dissemination of conspiracy theories	Rapid and widespread sharing of false information through social media platforms. Lack of fact-checking mechanisms allows misinformation to gain traction.	COVID-19 conspiracy beliefs mentioned 295,052 times in a single week on media platforms (cited in the study). High virality of misinformation compared to factual content.
Havey, 2020, USA [13]	Political ideology and polarization, social media echo chamber, populist rhetoric	Conservative users are more tolerant of misinformation. Facilitates consensus-seeking and amplifies misinformation. Promotes anti-science and conspiratorial narratives.	Conservatives dominate 5 of 6 misinformation topics, showing partisan spread of conspiracy theories. Twitter’s filter bubbles increase ideological reinforcement and misinformation spread. Conservatives reject public health guidelines, amplifying conspiracies through partisan media and political rhetoric.
Agley et al., 2021, USA [22]	Low trust in science, political orientation, perceived severity of COVID-19	Distrust in science and politicization of narratives amplify misinformation. Perceived severity of COVID-19 influences belief in conspiracy theories.	Statistical analysis shows a strong association between low trust in science and misinformation belief. Political orientation influences the likelihood of accepting conspiracy narratives.
Loomba et al., 2021, UK & USA [4]	Social media misinformation, sociopolitical influences, distrust of public health authorities	Misinformation spreads faster than factual content. Political polarization exacerbates distrust in public health authorities.	Misinformation exposure led to a measurable decline in vaccine intent. Sociopolitical factors like the 2020 US elections contributed to the spread of conspiracy beliefs.
Natoli & Marques, 2021, Australia [11]	Decreased trust in health authorities, feelings of powerlessness	Erodes confidence in medical recommendations. Amplifies skepticism towards pharmaceutical motives and public health guidelines.	Experimental studies showed reduced trust in health authorities following exposure to conspiracy theories. Feelings of powerlessness had varied influence on health-seeking behaviors.
Juanchich et al., 2021, UK [12]	Mistrust in government, reliance on social media, lower analytical thinking	Facilitates belief in misinformation and conspiracy theories. Lower analytical thinking increases vulnerability to misinformation.	Correlation between mistrust in government and conspiracy beliefs. Sharing of misinformation more frequent among conspiracy believers.
Dȩbski et al., 2022, Poland [7]	Belief in control of information, global conspiracies, personal well-being	Heightened information uncertainty, media contradictions, and anxiety about health and freedom. Increases reliance on conspiracy theories to explain uncertainties.	Correlation with belief in conspiracy theories (*r* = 0.768). Association with symptoms of anxiety and depression.
McCarthy et al., 2022, Australia [5]	Anomie, political ideology, perceived health threat	Increased perception of societal decline and reduced trust. Right-wing ideology associated with lower trust in government and higher conspiracy beliefs. Lower perceived health threat reduced need for vaccination.	Anomie was linked to greater vaccine hesitancy and acceptance of conspiracy beliefs. Bivariate correlations showed significant associations with vaccine hesitancy. Mediation models demonstrated how these factors influence hesitancy.
Jiang et al., 2022, Hong Kong [15]	Low political trust, viral spread of misinformation	Erodes trust in health advocacy and government, making individuals more susceptible. Higher influence of anti-vaccine messaging via social media platforms.	Distrust in Hong Kong government. Anti-vaccine messaging was highly influential through social media channels.
Swire-Thompson et al., 2023, USA [26]	Memory failures, disbelief in corrections, low depth of encoding	Memory failures lead to forgetting misinformation corrections, fostering belief regression. Shallow processing of corrective information limits long-term memory retention.	78% of participants who believed misinformation one month later misremembered corrections as affirmations. Evidence suggests that real-world corrections are processed more shallowly than experimental corrections.
Lin et al., 2023, multinational (66 countries) [16]	Social media proliferation, low trust in institutions, high HDI in wealthy nations	Social media amplified conspiracy theories. Distrust in government and science undermined compliance with public health measures.	Conspiracy endorsement was stronger in developed countries. Lower trust led to lower compliance with health interventions and public health guidelines.
Nefes et al., 2023, Spain [8]	Low trust in government health authorities, pharmaceutical companies, extreme right-wing ideology	Undermines belief in vaccine safety and adherence to public health guidelines. Fosters suspicion of vaccine development motives and misinformation acceptance. Promotes conspiratorial narratives against health policies and institutions aligned with opposing ideologies.	R-squared: 0.09 association with lower trust in health authorities correlating to conspiracy beliefs. R-squared: 0.24 significant negative association between trust in pharmaceutical companies and conspiracy beliefs. Participants identifying as extreme-right showed 0.35 SD greater conspiracy beliefs than centrists.
Walter et al., 2023, USA [25]	Politicization of science, use of scientific language by extremists, White nationalist ideology	Promotes distrust in scientific consensus, leveraging pre-existing partisan divides. Dresses conspiracy theories in scientific terms, making them appear credible. Connects conspiracy theories to racist and anti-Semitic narratives, spreading distrust in public health.	Conservative media and political figures like President Trump undermined health officials and science. Posts often discussed vaccine development and alternative medicine using pseudo-scientific language. Users described vaccines as tools for demographic control and labeled COVID-19 as a Jewish hoax.
Banas et al., 2024, USA [23]	Misinformation, emotional narratives, distrust in public health	Misinformation undermines decision-making. Emotional narratives reinforce beliefs and distrust amplifies skepticism toward public health.	References to anti-vaccination propaganda, particularly Andrew Wakefield’s fraudulent research and the documentary “Vaxxed.”
Kapoor et al., 2024, India [10]	Low socioeconomic status, right-leaning ideology, negative moral emotions	Creates a sense of powerlessness, endorses alternative narratives, and fosters mistrust in public health authorities.	Regression analysis highlighted predictors of conspiracy beliefs, including low socioeconomic status, right-leaning ideology, negative moral emotions, and mistrust in political institutions.
Lyons et al., 2024, USA [17]	False cancer beliefs, conspiracism, anti-expert sentiments, low digital and health literacy	False cancer beliefs, conspiracism, and anti-expert sentiments predispose individuals to endorse misinformation. Low digital and health literacy make it harder for individuals to evaluate the credibility of information.	False cancer beliefs strongly predicted perceived accuracy of inaccurate headlines and greater sharing intent for such misinformation. Strong correlation between low digital literacy and endorsement of inaccurate news headlines.
Carletto et al., 2024, USA [9]	Low education levels, medical mistrust, non-reliance on credible sources	Less ability to critically assess information, distrust in institutions increases conspiracy beliefs.	Lower education = 22% more likely to believe myths, higher mistrust = 72% more likely.
Kroke & Ruthig, 2024, USA & Canada [27]	Sociopolitical distrust, misinformation through social media, perceived lack of transparency	Undermines trust in health messages, promotes alternative narratives.	Strong correlation between endorsement of conspiracy theories and reduced vaccination.
Moran et al., 2024, USA [18]	Parasocial relationships, platform affordances, distrust in institutions, gendered narratives	Build trust through relatability and emotional connection; platform features like Instagram Stories amplify content.	Observations of influencers using personal anecdotes and religious narratives to gain trust and spread vaccine misinformation.

### How contributing factors spread conspiracy theories

The spread of health-related conspiracy theories is driven by a combination of historical, psychological, sociopolitical, and technological factors that reinforce skepticism and amplify misinformation. Historical mistrust, particularly among minority populations, stems from events like the Tuskegee Study and continues to undermine confidence in public health initiatives. This mistrust is more pronounced among individuals with lower education and income levels [2]. Additionally, sociocultural factors such as religious beliefs, reliance on CAM, and poor mental health contribute to conspiracy thinking by fostering skepticism toward biomedical science [1, 21, 24].

The role of the media—both alternative and social—has been pivotal in spreading misinformation. During the Zika outbreak, the absence of verified content allowed alternative narratives to dominate through emotional appeal and repetition [14]. Similarly, frames emphasizing government collusion or pharmaceutical profiteering make false claims more likely to be believed and shared [3, 6]. Social media platforms act as echo chambers to reinforce pre-existing biases and promote conspiratorial narratives, particularly among conservative users who exhibit greater tolerance for misinformation [13, 22].

Misinformation spreads rapidly, often outpacing corrections. False claims about vaccines, for example, circulate faster than verified information [4, 11]. Contradictions in media reporting further amplify anxiety, leading individuals to seek explanations that align with their pre-existing beliefs [7]. Political ideology, particularly right-wing perspectives, and concerns about societal decline further intensify distrust in government and support for global conspiracy narratives [5, 8].

Cognitive biases and information-processing limitations contribute to the persistence of conspiracy beliefs. Memory failures, shallow engagement with corrective information, and belief regression—where individuals revert to misinformation even after being exposed to fact-checks—hinder efforts to counter false narratives [26]. Low digital and health literacy exacerbate this issue by reducing individuals’ ability to assess the credibility of information, increasing their vulnerability to conspiracy-driven content [9, 17].

Distrust in health systems and scientific institutions is further reinforced by political divisions, emotional messaging, and misinformation disguised in scientific language. Some conspiracy theories incorporate pseudoscientific terminology to appear credible, while others leverage racist or anti-Semitic undertones to delegitimize public health policies [25]. Emotional narratives, particularly those portraying vaccines as harmful or health authorities as corrupt, deepen skepticism and resistance to medical interventions [10, 23]. On social media, influencers use platform features such as Instagram Stories to establish emotional connections with their audience, further amplifying distrust in traditional health sources [18].

### Empirical evidence on factors driving conspiracy beliefs

Empirical evidence demonstrates how various factors contribute to the spread of conspiracy theories in health. Mistrust in government and systemic inequalities are consistently linked to conspiracy beliefs, particularly among minority populations. In the United States, conspiracy beliefs were found to be over three times more common among Black and Hispanic individuals compared to White populations, with lower education and income levels serving as strong predictors [2]. Similarly, high levels of mistrust (72%) were associated with reduced HIV testing rates, illustrating the direct behavioral impact of these beliefs [1]. In South Africa, the legacy of AIDS denialism under Thabo Mbeki’s leadership delayed the rollout of antiretroviral therapy, fostering distrust and reinforcing reliance on non-evidence-based explanations, particularly among adolescents in Soweto [21].

The influence of misinformation and alternative media is substantial. During the Zika virus outbreak, 66% of widely shared news stories came from alternative media sources, with misinformation spreading more rapidly than verified content. Delays in fact-checking efforts from organizations like the WHO exacerbated this issue [14]. On social media, trolls and automated bots amplified anti-vaccine messages [3]. In Ecuador, an analysis of online discourse identified over 295,052 mentions of COVID-19 conspiracy theories within just one week [6]. In the US, conservative users dominated five of six major misinformation topics, leveraging ideological filter bubbles to reject public health guidelines [13].

Sociopolitical factors further influence the acceptance of conspiracy beliefs. Misinformation during the 2020 US elections led to measurable declines in vaccine intent, highlighting the intersection of political polarization and health misinformation [4]. Experimental studies showed that exposure to conspiracy theories significantly reduced trust in health authorities, with feelings of powerlessness playing a mediating role in health-seeking behaviors [11]. In Poland, a correlation as high as 0.768 was found between conspiracy beliefs and symptoms of anxiety and depression [7]. In Australia, perceptions of societal decline and anomie contributed to vaccine hesitancy through increased conspiracy endorsement [5].

Cognitive factors also reinforce the persistence of conspiracy beliefs. In a US study, 78% of individuals who initially believed misinformation later misremembered corrections as confirmations, highlighting the challenges of countering falsehoods [26]. On a global scale, conspiracy beliefs were stronger in developed nations, where lower trust in institutions was associated with reduced compliance with public health measures [16]. In Spain, distrust in pharmaceutical companies and extreme-right political ideology were significant predictors of conspiracy beliefs, with individuals identifying as extreme-right exhibiting 0.35 standard deviations higher conspiracy belief levels compared to centrists [8].

The role of social and emotional narratives in spreading conspiracy theories is significant. In the Unites States, conservative media and political figures, including President Trump, actively undermined trust in public health officials. Pseudo-scientific language was also employed to legitimize alternative medicine and conspiratorial claims, including allegations that COVID-19 was a “Jewish hoax” [25]. On social media, influencers used personal anecdotes and religious narratives to gain trust and disseminate vaccine misinformation [18]. In India, regression analysis identified low socioeconomic status, right-leaning ideology, and negative moral emotions as significant predictors of conspiratorial beliefs [10].

Low literacy levels exacerbate susceptibility to conspiracy theories. Individuals with lower education levels were 22% more likely to believe in myths, while those with institutional mistrust had a 72% higher likelihood of endorsing conspiracy beliefs [9]. Misinformation about cancer treatments, paired with low digital literacy, was strongly correlated with both the perceived accuracy and the sharing of inaccurate health-related headlines [17]. Studies have consistently linked reduced vaccination intent with the influence of conspiracy beliefs, highlighting the broad impact of these narratives on public health decision-making.

### Strategies to counter health-related conspiracy theories

Efforts to counter the spread of conspiracy theories in health focus on education, trust-building, and strategic communication. Community engagement and culturally relevant educational initiatives play a crucial role in addressing misinformation, particularly among marginalized groups. Targeted campaigns have been developed to promote trust in public health institutions and correct misconceptions among specific populations, such as minorities in the United States and adolescents in South Africa [1, 2, 21] (Table 4).

Educational interventions emphasize the importance of early, accurate information and collaboration with fact-checkers to prevent misinformation from gaining traction. For example, during the Zika outbreak, early exposure to credible information and partnerships with fact-checking organizations were recommended to counter the dominance of alternative media narratives [14]. Visual tools such as infographics explaining the scientific process have been shown to enhance trust in science and reduce susceptibility to misinformation [22]. Additionally, vaccination campaigns incorporating altruistic messaging and structured fact-checking initiatives have demonstrated effectiveness in mitigating conspiracy-driven vaccine hesitancy [4].

Tailored communication strategies targeting populations skeptical of modern medicine have proven effective in improving understanding and trust. Techniques such as two-sided refutational messaging, which presents misinformation before debunking it, have been successfully employed to counter conspiracy narratives [3, 15]. Promoting analytical thinking, fostering transparent dialogue, and addressing logical fallacies within conspiracy beliefs are critical to enhancing public trust in health recommendations [12, 27].

Cognitive-based interventions such as inoculation treatments—both fact-based and logic-based—have been shown to build resistance to misinformation. Strategies that incorporate repeated corrections and deeper cognitive engagement with the facts help overcome psychological barriers to belief revision [23, 26]. Strengthening critical thinking skills and institutional trust is particularly vital in societies with low political or scientific trust [10].

Social media regulation and policy interventions are essential in curbing the spread of misinformation. Suggested measures include suspending accounts that propagate false health claims, limiting external linking features to unreliable sources, and strengthening advertisement guidelines for health-related products [13, 18]. Monitoring extremist forums to identify and counter the misuse of pseudo-scientific language in conspiracy narratives has also been proposed as a preventative measure [25].

Enhancing health literacy and promoting reliance on credible sources form the foundation of long-term misinformation mitigation. Public education on vaccine development, tailored debunking efforts, and encouraging trust in medical professionals and government agencies help bolster resilience against misinformation [9]. Specific media literacy interventions, such as BOAST (health-focused) and broader news literacy training, have demonstrated promise in improving individuals’ ability to discern facts from misleading headlines [17].

**Table 4:** Strategies to address conspiracy beliefs

Author(s), year, country	Strategies to counter health-related conspiracy theories	How strategies address and mitigate conspiracy beliefs	Effectiveness of the strategies	Evidence-based approaches to countering conspiracy beliefs
Russell et al., 2011, USA [2]	Community engagement, trust-building, education campaigns	Involves communities in research to address mistrust and improve health literacy	Proposed but not directly tested	Indirect evidence supports engagement as a potential intervention strategy
Ford et al., 2013, USA [1]	Addressing misconceptions, improving trust in government	Reduces psychological barriers to healthcare engagement	Not explicitly tested	Alternative strategies for engaging distrustful individuals
Hogg et al., 2017, South Africa [21]	Culturally relevant health education	Increases understanding of HIV origins among adolescents	Not explicitly measured	Highlights the need for culturally tailored approaches
Lahrach & Furnham, 2017, UK [24]	Tailored communication for skeptical patients	Improves patient satisfaction and adherence to medical advice	Hypothesized but not directly evaluated	Suggests a need for healthcare providers to better understand patient beliefs
Sommariva et al., 2018, USA [14]	Early exposure to accurate information, fact-checking, risk communication	Limits misinformation spread and enhances message accuracy	WHO interventions and Facebook’s algorithm adjustments reduced misinformation	WHO’s “Dispelling Rumors Around Zika” and Facebook’s fact-checking strategies
Featherstone & Zhang, 2020, USA [3]	Two-sided refutational messaging	Refutes false claims, reducing anger and increasing vaccine acceptance	Demonstrated improved pro-vaccine attitudes	Supported by controlled trials
Chen et al., 2020, Ecuador [6]	Web-based psychiatric screening, targeted health messaging	Identifies individuals with high conspiracy beliefs and provides corrective information	Stated as critical but not directly measured	Supports the role of social media in combating misinformation
Havey, 2020, USA [13]	Suspending misinformation accounts, educating on source credibility	Limits misinformation amplification and improves information discernment	Not explicitly reported	Highlights inconsistencies in social media misinformation policies
Agley et al., 2021, USA [22]	Infographics on scientific processes	Enhances trust in science, reducing misinformation beliefs	Small but statistically significant improvement	Randomized controlled trial confirms effectiveness
Loomba et al., 2021, UK & USA [4]	Altruistic vaccination messaging, fact-checking misinformation	Increases vaccine uptake by emphasizing community protection	Higher vaccination intent in groups exposed to altruistic messaging	Survey results show factual exposure increases vaccine willingness
Natoli & Marques, 2021, Australia [11]	Evidence-based counter-narratives	Decreases belief in conspiracy theories and improves trust in health authorities	Effective in reducing conspiracy beliefs compared to control groups	Highlights the role of counter-narratives.
Juanchich et al., 2021, UK [12]	Analytical thinking promotion, transparent communication	Encourages critical thinking	Analytical thinking lowered misinformation acceptance	Experimental results validate effectiveness
Dȩbski et al., 2022, Poland [7]	WHO-led myth debunking, fact dissemination	Strengthens trust in health information, reduces false beliefs	Promotes mental health and compliance with health measures	WHO’s COVID-19 myth-busting strategies cited
McCarthy et al., 2022, Australia [5]	Trust-building in government, community engagement, misinformation countering	Enhances institutional trust and reduces misinformation spread	Higher trust linked to vaccine acceptance and lower conspiracy endorsement	Suggested as a key strategy for reducing vaccine hesitancy
Jiang et al., 2022, Hong Kong [15]	Inoculation messaging (two-sided refutation)	Builds resistance to rumors, improving vaccine attitudes	More effective than supportive messaging	Experimental evidence supports inoculation messaging
Swire-Thompson et al., 2023, USA [26]	Repetition of corrections, deep encoding strategies	Improves memory retention and reduces belief regression	Correction memory accounts for 66% of belief regression variance	Repetition and active engagement enhance correction
Lin et al., 2023, multi-national (66 countries) [16]	Tailored public health campaigns, risk communication	Strengthens public trust and mitigates misinformation through targeted messaging	Emphasized as necessary but not directly tested	Evidence shows high conspiracy belief correlates with lower trust in health guidelines
Nefes et al., 2023, Spain [8]	Trust-building in science, targeted messaging to extreme-right groups	Increases trust in health authorities and reduces ideological misinformation	Transparency and scientific literacy programs lowered skepticism	Higher trust correlated with lower conspiracy belief scores
Walter et al., 2023, USA [25]	Monitoring extremist forums	Identifies and tracks misinformation narratives	Suggested for public health and law enforcement use	Provides insights into misinformation trends and behaviors
Banas et al., 2024, USA [23]	Fact-based and logic-based inoculation treatments	Equips individuals to resist anti-vaccine misinformation	Both methods effectively counter conspiracy theories	Fact-based refutation addresses misinformation, logic-based inoculation corrects reasoning errors
Kapoor et al., 2024, India [10]	Strengthening critical thinking, institutional trust	Reduces misinformation susceptibility, promoting evidence-based health practices	Theoretical framework, requires further empirical research	Suggested as a promising intervention strategy
Lyons et al., 2024, USA [17]	Media literacy training (News Tips), health misinformation intervention (BOAST)	Encourages skepticism toward misleading headlines, improving critical news evaluation	Reduced intent to share false health news	BOAST intervention had mixed effects, indicating need for refinement
Carletto et al., 2024, USA [9]	Promoting reliance on credible sources	Strengthens trust in healthcare and reduces misinformation spread	Participants relying on medical professionals were 28% less likely to believe conspiracy myths	Statistical analysis supports credibility-based interventions
Kroke & Ruthig, 2024, USA & Canada [27]	Vaccine education, addressing conspiracy fallacies	Reduces fear, enhances risk perception, counters misinformation	Most effective when introduced early	Supports transparency and proactive education strategies
Moran et al., 2024, USA [18]	Restricting misinformation-linked external features, FTC regulations on health ads	Limits financial incentives for misinformation	Not directly tested	Suggests stricter advertising guidelines and platform monitoring measures

### How strategies address and mitigate conspiracy beliefs

Efforts to mitigate conspiracy beliefs target their underlying causes while encouraging trust in public health and evidence-based information. Community engagement and educational initiatives address systemic mistrust by involving communities in research, improving public understanding of health policies, and reducing psychological barriers to behaviors such as HIV testing [1, 2]. In South Africa, educating adolescents about the true origins of HIV has reduced reliance on conspiracy narratives, strengthening their understanding of biomedical science [21].

Tailored communication and misinformation correction play a crucial role in countering conspiracy beliefs. Two-sided refutational messages, which present false claims alongside factual rebuttals, have been effective in reducing emotional resistance and improving attitudes toward vaccines and other health measures [3, 15]. Targeted health communication campaigns, particularly those designed for vulnerable populations, enhance the ability to distinguish credible information from misinformation, as demonstrated in Ecuador and other regions [6, 13]. Social media interventions, including flagging false content and limiting the reach of conspiracy theories, encourage self-correction among users and decrease the visibility of harmful narratives [14, 18].

Building trust in science and transparent communication are critical in reducing dependence on conspiracy beliefs. Strategies such as transparent government actions, fair policymaking, and proactive misinformation responses have been effective in countering perceptions of social instability and distrust in Australia [5]. Infographics explaining the scientific process have been shown to improve public confidence in science, indirectly decreasing susceptibility to misinformation [22]. Likewise, using altruistic messaging in vaccination campaigns—emphasizing the role of vaccines in protecting others—has led to increased vaccine uptake [4].

Memory-focused and cognitive strategies enhance the retention of accurate information while countering belief regression. Repeated corrections and encouraging deeper cognitive processing help individuals internalize accurate narratives, reducing the long-term effects of misinformation [23, 26]. Media literacy interventions that teach individuals to critically evaluate sensationalist health headlines have also been effective in lowering the spread and acceptance of misinformation [17].

Targeted interventions that address ideological divides and distrust in health institutions help strengthen adherence to public health measures. In Spain, reinforcing trust in health policies has contributed to increased vaccine acceptance [8]. Monitoring and countering conspiracy narratives in extremist online communities enable early intervention before misinformation escalates [25]. Providing accessible, evidence-based information has improved trust in healthcare systems, particularly among populations with historically low health literacy or high levels of skepticism [9].

These strategies work collectively to reduce fear, correct misinformation, and strengthen risk perception. By integrating community engagement, tailored communication, and transparent policymaking, these approaches mitigate conspiracy beliefs and promote trust in evidence-based healthcare practices [10, 27].

### Evaluating the effectiveness of strategies against conspiracy beliefs

The effectiveness of these strategies varies. Some showed promise while others remain theoretical or untested. Early interventions in rumor control and algorithmic adjustments on platforms like Facebook reduced the reach of misinformation while increasing the visibility of accurate health messages, particularly during the Zika outbreak [14]. Two-sided refutational messages significantly improved pro-vaccination attitudes compared to exposure to misinformation alone [3]. Inoculation strategies also showed success, with targeted messaging in Hong Kong proving more effective in increasing vaccine intentions and positive attitudes than supportive messages alone [15].

Trust-building strategies produced mixed but encouraging results. Infographics explaining the scientific process contributed to a small yet significant increase in trust, indirectly reducing susceptibility to misinformation [22]. Transparent communication and scientific literacy initiatives enhanced vaccination rates and reduced skepticism, particularly in regions with strong extreme-right political alignment, such as Spain [8]. In Australia, fostering trust in government and addressing perceptions of social instability were associated with higher vaccine acceptance and lower endorsement of conspiracy theories [5]. Additionally, individuals who relied on credible sources, such as medical professionals, were 28% less likely to believe conspiracy theories, reinforcing the importance of trusted information channels [9].

Cognitive and analytical strategies also yielded measurable benefits. Memory-focused interventions, such as repeated corrections and deeper cognitive encoding of accurate information, accounted for 66% of the variance in belief regression, highlighting their effectiveness in reducing misinformation retention [26]. Analytical thinking interventions in the UK decreased susceptibility to misinformation while increasing trust in public health recommendations [12]. However, media literacy interventions had mixed outcomes; while they significantly reduced the intent to share inaccurate news, they also heightened skepticism toward accurate information [17].

Some strategies remain largely theoretical or require further evaluation. While community engagement, tailored communication, and interventions aimed at vulnerable populations are considered essential, they have not yet been widely assessed for effectiveness [2, 6, 10]. Theoretical frameworks, including the use of law enforcement and public health agencies to counter misinformation and extremism, require empirical validation to determine their practical impact [25].

### Evidence-based approaches to countering conspiracy beliefs

The supporting evidence for strategies to mitigate conspiracy beliefs highlights their potential to counter misinformation and rebuild public trust. Community engagement and culturally tailored education have been identified as key interventions, emphasizing the importance of using alternative venues and culturally appropriate messaging to reach populations with high levels of mistrust [1, 2, 21]. Additionally, improving healthcare providers’ understanding of patient beliefs has been recommended to enhance communication and encourage compliance with public health measures [24].

The effectiveness of targeted messaging is demonstrated through initiatives such as the WHO’s “Dispelling Rumors Around Zika” campaign and Facebook’s efforts to amplify fact-checked information, which increased the visibility of accurate content while reducing the spread of misinformation [14]. Controlled experiments have further validated the impact of two-sided refutational messages in decreasing emotional resistance and improving attitudes toward vaccines. Meanwhile, web-based psychological screening has emerged as a promising approach to addressing conspiracy-related anxiety [3, 6].

Experimental studies support interventions such as inoculation messages, which strengthen resistance to misinformation by preemptively exposing individuals to flawed reasoning in conspiratorial narratives. A study in Hong Kong demonstrated that groups who receive inoculation messages showed significantly higher vaccine attitude scores than controls [15]. Similarly, randomized trials found substantial improvements in trust and vaccination intent when accurate information was combined with altruistic messaging, increasing vaccine willingness by 6.4% in the US and by 63.7% in the UK among those motivated by protecting others [4, 22].

Analytical thinking prompts and memory-focused strategies have been shown to reduce susceptibility to misinformation. Engaging individuals in active cognitive processing, such as careful reading and repetition of corrections, enhances information encoding and reduces belief regression over time [12, 26]. Media literacy interventions like News Tips decreased the intent to share inaccurate cancer-related news by 9% points, although findings from the BOAST intervention indicated the need for refinement, as it also increased skepticism toward accurate information [17].

Building trust in health authorities and ensuring transparency in public health communication have been linked to lower conspiracy belief scores, with each unit increase in trust corresponding to a 0.14 standard deviation decrease in conspiracy beliefs [8]. Efforts to engage with extreme-right political groups using transparency strategies have been identified as particularly effective in reducing skepticism and improving vaccination rates [27].

Stricter advertising guidelines and enhanced platform monitoring have been emphasized as necessary measures to limit the amplification of conspiracy narratives through external linking features and pseudo-scientific content. These strategies are essential for addressing the financial incentives that drive misinformation sharing on platforms such as Instagram [18, 25].

## Discussion

This scoping review highlights the widespread prevalence of health-related conspiracy theories, which have been shaped by complex sociocultural, psychological, and political factors. Historical discrimination and systemic mistrust among racial and ethnic minorities contribute significantly to HIV-related conspiracy beliefs, directly impacting health behaviors and intervention efforts [1, 2]. In South Africa, conspiracy beliefs among adolescents are associated with skepticism toward biomedical prevention and treatment, emphasizing the need for culturally tailored educational programs to address misinformation [21]. The role of health beliefs and misinformation as key drivers of conspiracy endorsement further emphasizes the necessity of targeted interventions, particularly those addressing structural inequities [24]. As misinformation spreads, conspiracy theories do not remain static; they evolve in response to major crises, political shifts, and emerging health threats. Narratives from past public health crises, such as HIV/AIDS, are often repurposed and adapted to new contexts like the COVID-19 pandemic, fueling persistent mistrust and misinformation. This cyclical pattern reinforces skepticism toward healthcare systems and amplifies resistance to public health interventions.

Beyond fostering skepticism, conspiracy theories have substantial behavioral, psychological, and social consequences that hinder public health efforts. Exposure to conspiracy narratives reduces trust in health authorities, leading to lower vaccination intent and decreased engagement with preventive healthcare services [4, 11]. Additionally, conspiracy beliefs are linked to increased anxiety, depression, and vaccine hesitancy, further exacerbating their psychological toll [5, 7]. These mental health impacts can contribute to avoidance of healthcare services, reinforcing distrust in health institutions. Political ideology and social media play a role in amplifying misinformation, reinforcing ideological polarization, and resistance to public health measures [8, 13]. Marginalized populations, particularly those with lower socioeconomic status or limited health literacy, are disproportionately affected by these beliefs, creating significant barriers to accessing accurate health information and care [8, 13]. This highlights the inequitable burden of conspiracy theories on already vulnerable communities, further widening health disparities.

Digital platforms speed the dissemination of conspiracy theories, often outpacing verified health information. Misinformation on social media spreads widely due to algorithmic amplification, further fueling distrust in scientific expertise [6, 14]. The emotional appeal of conspiracy narratives, particularly those invoking anger and fear, enhances their persuasiveness and longevity [3]. These findings emphasize the need for proactive interventions, including content moderation, fact-checking initiatives, and regulatory measures such as misinformation flagging and improved content verification to complement educational strategies [18, 25].

While several strategies to mitigate conspiracy beliefs have been proposed, their effectiveness varies across different contexts. Inoculation messages and logic-based refutations have shown promise in countering misinformation, particularly in vaccine-related conspiracies [15, 23]. Repeated corrective messaging and efforts to build public trust in science contribute to reducing belief regression and misinformation acceptance [22, 26]. Among the interventions examined, inoculation messaging has shown the strongest evidence for improving vaccine-related attitudes in low-trust environments [17], whereas fact-based refutations were more effective at countering misinformation but had limited spillover protection against unrelated conspiracies [16]. Media literacy interventions showed mixed results; while they improved critical evaluation skills, they also heightened skepticism toward accurate health information [18]. This suggests that while each intervention has merit, their effectiveness depends on the sociopolitical context and target population. Addressing the social determinants of health, such as education, socioeconomic disparities, and cultural barriers, is critical in reducing the susceptibility of underserved populations to conspiracy theories [5, 27]. Interventions must account for these inequities to ensure that solutions are inclusive and equitable. However, interventions must be tailored to specific sociopolitical environments, as addressing ideological divides and promoting critical thinking are essential for combating misinformation effectively [10, 16]. Despite identifying multiple interventions to counter conspiracy beliefs, there remains a critical gap in comparative evaluations. No studies have directly compared the relative effectiveness of different countermeasures, making it difficult to determine which interventions work best in specific sociopolitical contexts or among different populations. This gap limits the ability to develop evidence-based, scalable solutions. Future research should prioritize head-to-head comparisons of intervention strategies, particularly in marginalized communities, where misinformation has the greatest impact.

In addition to existing interventions, several emerging strategies warrant further exploration in combating health conspiracy theories. Credibility labels, both peer-supplied and platform-driven, have been suggested as a means to enhance the visibility of accurate health information while reducing the reach of conspiracy-based misinformation [28, 29]. However, their long-term effectiveness remains uncertain, particularly among individuals already distrustful of authoritative sources. Research suggests that while credibility labels improve users’ ability to differentiate between reliable and unreliable health information, their overall impact on reducing conspiracy beliefs related to vaccines and public health policies is limited [28]. Automated content moderation, using artificial intelligence to detect and remove flagged misinformation, presents another promising but controversial approach. While AI-driven moderation can help curtail the spread of misleading health narratives, concerns remain regarding biases and potential over-censorship, particularly in discussions about vaccine safety, alternative treatments, or governmental public health measures [30]. Demonetization efforts, aimed at restricting revenue streams for conspiracy theorists and misinformation actors, have been introduced on some platforms, yet their effectiveness in curbing widespread conspiracy narratives remains uncertain. Many conspiracy networks adapt by migrating to alternative platforms or leveraging crowdfunding, limiting the long-term impact of demonetization [31]. Prebunking strategies, including gamification-based misinformation resistance, have shown promise in controlled settings by helping individuals develop cognitive resistance to misleading conspiracy claims [32]. Studies suggest that inoculation theory-based prebunking can be particularly effective in countering vaccine-related conspiracy theories and COVID-19 misinformation, but further research is needed to evaluate its adaptability across different sociopolitical and cultural contexts, particularly in marginalized communities where distrust in public health institutions is deeply rooted. While these emerging strategies offer potential solutions, their effectiveness in mitigating health conspiracy theories remains an open question, emphasizing the need for sustained, evidence-based interventions tailored to diverse populations.

Significant research gaps remain regarding the long-term effectiveness of counter-misinformation strategies. While the spread and impact of health-related conspiracy theories have been extensively documented, few studies have assessed the value of mitigation efforts. Many proposed interventions, including media literacy programs and trust-building initiatives, lack rigorous testing in diverse populations [9, 17]. Populations in underserved or resource-constrained settings are especially vulnerable, as conspiracy beliefs further hinder access to essential healthcare services, perpetuating public health disparities. Tackling these inequities requires sustained efforts to engage communities, build trust, and bridge gaps in healthcare access [5, 27]. The persistence of deep-seated mistrust, particularly among marginalized communities, complicates efforts to implement lasting solutions [5, 27]. Addressing these challenges requires a combination of evidence-based public health communication, community engagement, and digital regulation. Future research should prioritize large-scale, long-term studies to assess misinformation countermeasures and explore innovative methods to rebuild trust in health systems, particularly for vulnerable populations.

This review highlights the importance of culturally sensitive messaging, transparent health communication, and digital interventions in limiting the spread of misinformation. Future efforts should focus on evaluating the effectiveness of mitigation strategies and adapting them to different sociopolitical contexts. Policymakers, educators, and healthcare organizations can use these findings to develop interventions that strengthen public trust, improve media literacy, and enhance misinformation detection strategies across diverse communities.

### Limitations

This study has several limitations. First, it relied exclusively on primary research articles published in peer-reviewed journals, which may have excluded valuable insights from gray literature, government reports, and expert analyses. Second, the review only included studies written in English, potentially missing important research conducted in non-English-speaking countries. Given the global nature of conspiracy theories, insights from other linguistic and cultural contexts could have provided a more comprehensive understanding of the issue. Third, while this review identified various interventions aimed at mitigating conspiracy beliefs, there was a lack of comparative studies on the effectiveness of different approaches across diverse populations. Without direct comparisons, it remains unclear which strategies are most effective in different sociopolitical and cultural settings, which limits the ability to provide concrete policy recommendations. Additionally, some interventions may have unintended consequences, such as reinforcing skepticism among certain groups or increasing resistance to corrective messaging. Fourth, many of the included studies relied on self-reported data, which may be subject to bias, including social desirability and recall inaccuracies. This is particularly relevant in studies examining sensitive topics such as mistrust in health authorities and vaccine hesitancy. Fifth, the role of algorithmic bias in amplifying misinformation on social media was not explicitly addressed in most studies. The selective exposure created by digital platforms may distort public perception and further entrench conspiracy beliefs, limiting the effectiveness of countermeasures. Sixth, most of the reviewed studies focused on Western and high-income countries, which may not fully capture the experiences of populations in low- and middle-income settings. Factors such as political instability, healthcare access, and historical distrust in government institutions vary across regions, making it essential for future research to examine how conspiracy beliefs manifest in different geopolitical contexts. Seventh, the review did not assess the long-term effectiveness of interventions designed to counter conspiracy beliefs. While some strategies, such as inoculation messages and media literacy programs, showed promise, there is limited evidence on their effectiveness over time. More longitudinal studies are needed to determine whether these interventions lead to lasting changes in attitudes and behaviors. Eighth, the impact of misinformation corrections on deeply entrenched beliefs remains uncertain. Some studies suggest that repeated exposure to debunking efforts may inadvertently reinforce false beliefs in certain groups, a phenomenon known as the “backfire effect.” This highlights the need for more nuanced research into the psychological mechanisms that shape misinformation retention and resistance to correction. Finally, while this review synthesized key findings on the spread and impact of health-related conspiracy theories, it did not analyze how these beliefs evolve over time, particularly in response to major events such as pandemics or political crises. Future research should explore the dynamic nature of conspiracy beliefs to develop more adaptive and effective strategies for countering them.

## Conclusion

This scoping review highlights the pervasive and detrimental effects of conspiracy theories in the health sector. These theories, fueled by mistrust in governments, public health authorities, and scientific institutions, undermine critical health initiatives such as vaccination programs and compliance with public health measures. They thrive on misinformation, social media amplification, and emotional narratives, disproportionately impacting vulnerable groups, including those with lower socioeconomic status and limited health literacy. These inequities are particularly evident in marginalized communities, where historical injustices and systemic barriers exacerbate the harmful effects of conspiracy beliefs on access to care and health outcomes. The consequences of these theories emphasize the need for targeted interventions to address their root causes and mitigate their influence.

Actionable strategies must prioritize trust-building through transparency and consistent engagement with communities. Public health campaigns should leverage culturally relevant and emotionally resonant messaging to counter the appeal of conspiracy theories. Equally, educational initiatives aimed at improving media literacy and critical thinking can equip individuals to discern misinformation. Collaborative efforts between policymakers, healthcare professionals, educators, and social media platforms are essential to curb the dissemination of harmful narratives and promote accurate health information. Strengthening regulatory measures to counter misinformation while maintaining freedom of expression is another key policy consideration.

Moving forward, addressing health-related conspiracy theories requires a proactive and multifaceted approach. Investments in research to understand the psychological and social drivers of these beliefs can guide the design of more effective interventions. Additionally, acknowledging the challenges of intervention strategies, such as resistance to debunking efforts and the potential for unintended consequences, is crucial for refining future approaches. Integrating these insights into public health strategies will be pivotal in restoring trust, improving health outcomes, and building resilience against future waves of misinformation.

Recent policy shifts by major social media platforms have weakened misinformation moderation, exacerbating the challenge of combating health-related conspiracy theories. The divestment of content moderation teams by X (formerly Twitter), Meta’s reduced reliance on third-party fact-checkers, and Google’s rejection of the European Union’s (EU) fact-checking commitments contradict the findings of this review, which highlight the need for stronger regulatory oversight and proactive misinformation mitigation strategies [33]. The weakening of platform-driven countermeasures risks allowing conspiracy theories to spread unchecked, further undermining public trust in health communication. In response, alternative regulatory actions—such as the EU’s formalization of the Code of Practice on Disinformation under the Digital Services Act (DSA)—alongside independent initiatives led by non-governmental organizations (NGOs), educators, and researchers, will play an increasingly critical role in countering the impact of misinformation [34].

Future research should examine how conspiracy theories adapt to public health crises and evaluate real-time countermeasures to combat misinformation effectively. As social media policies increasingly allow unregulated misinformation to flourish, public health strategies must adapt by leveraging cross-sector collaborations, policy interventions, and community engagement efforts. Safeguarding communities against health conspiracy theories requires a proactive, evidence-based approach that integrates digital regulation, public trust-building, and global health resilience strategies.

## Data Availability

No datasets were generated or analysed during the current study.
